# Supplementary material for: Health system costs and days in hospital for colorectal cancer patients in New South Wales, Australia
Source: PLoS One. 2021 Nov 29;16(11):e0260088. doi: 10.1371/journal.pone.0260088 (PMC8629237; doi:10.1371/journal.pone.0260088)
Supplement: Attachment — Submitted filename: S1_File.docx [file pone.0260088.s003.docx]

**Health system costs and days in hospital for colorectal cancer patients in NSW, Australia**

**Supporting information**

*Table A. Codes/item numbers for identifying colorectal cancer treatment with surgery, chemotherapy, or radiotherapy.*

| **Treatment type** | **Data type** | **Codes/Item numbers** |
| --- | --- | --- |
| Surgery | APDC procedure (including all sub-codes) | 32000,32003-32015,32024-32026,32028,32030,32033,32039,32042,32045-32047,32051,32099,32102-32106, 32108,32112,90308,90315,90341,90450,90959 |
|  | MBS item | 32000,32003-32015,32024-32026,32028,32030,32033,32039,32042,32045-32047,32051,32099,32102-32106, 32108,32112 |
| Chemotherapy | APDC procedure (including all sub-codes) | 13915-00,13918-00,13921-00,13924-00,13927-00,15304,15312,15319,15327,15338,15360,90760-00,90767-00, 90768-00,96199-00,96199-09,96200-00,96201-00, 96203-00, 96204-00 |
|  | APDC diagnosis | Z51.1, Z51.2 |
|  | MBS item | 13915,13918,13921,13924,13927,13930,13933,13936,13945 |
|  | PBS ATC code | All codes starting with “L01” (L01AA01-L01XX41) except L01BA01 (methotrexate, more likely for rheumatoid arthritis) |
| Radiotherapy | APDC procedure | 15100-00,15203-00,15204-00,15207-00,15208-00,15224-00,15239-00,15254-00,15269-00,15506-01,15506-02, 15518-00,15521-00,15524-00,15550-00,15600-00,15600-01,90765-00,90765-01,90765-02,90765-03 |
|  | APDC diagnosis | Z51.0 |
|  | MBS item | 15000-15399, 15500-15600, 15700-15899 |

*Note: Records included were from 30 days prior to cancer registry diagnosis date onwards.*

*APDC: Admitted Patient Data Collection; ATC: Anatomical Therapeutic Chemical Classification System; MBS: Medicare Benefits Schedule; PBS: Pharmaceutical Benefits Scheme.*

Targeted therapies and immunotherapy

Among colon cancer cases, 10% had bevacizumab and 3% had cetuximab. For rectal cancer, the proportions were 12% and 4%, respectively. The largest proportions by year of diagnosis for all CRC cases given targeted therapies were 16% and 6%, respectively, both for cases diagnosed in 2010, with virtually no records of these medications for cases diagnosed before 2008 (prior to their approval for subsidisation). Almost 30% of cases with metastatic disease at diagnosis had bevacizumab, compared with 10% of regional cases and 2% of localised cases. Only a few cases had panitumumab, and there were no records of any other monoclonal antibodies being used. Of the angiogenesis inhibitors, 1% of cases each year had a record of aflibercept and there were no records of regorafenib use, nor were there any records of the immunotherapies pembrolizumab or nivolumab, as these were not listed for subsidisation during the study period.

*Table B. Descriptive statistics for included colon and rectal cancer cases diagnosed 2006-2013, compared with all colon and rectal cancer cases diagnosed in NSW.*

|  | **Colon cases (n=1200)** | | **Rectal cases (n=546)** | | **All NSW^a^** | |
| --- | --- | --- | --- | --- | --- | --- |
|  | **No. of cases** | **% of cases** | **No. of cases** | **% of cases** | **Colon cases** | **Rectal cases** |
| Stage at diagnosis |  |  |  |  | *2011-2015* | |
| Localised | 398 | 33% | 187 | 34% | 30% | 33% |
| Regional | 492 | 41% | 221 | 40% | 44% | 38% |
| Distant metastases | 243 | 20% | 90 | 16% | 19% | 17% |
| Unknown | 67 | 6% | 48 | 9% | 8% | 13% |
| Age at diagnosis (years) |  |  |  |  | *2013-2015* | |
| Median age (inter-quartile range) | 72 (65-80) |  | 68 (61-76) |  | 72 | 68 |
| 45-54 | 62 | 5% | 58 | 11% | 7%^b^ | 12%^b^ |
| 55-64 | 220 | 18% | 147 | 27% | 16% | 23% |
| 65-74 | 417 | 35% | 183 | 34% | 29% | 29% |
| ≥75 | 501 | 42% | 158 | 29% | 43% | 30% |
| Sex |  |  |  |  | *2013-2015* | |
| Female | 624 | 52% | 191 | 35% | 50% | 40% |
| Male | 576 | 48% | 355 | 65% | 50% | 60% |
| Remoteness of place of residence |  |  |  |  | *2014-2016* | |
| Major cities | 630 | 53% | 256 | 47% | 67% | 68% |
| Inner regional | 413 | 34% | 210 | 38% | 24% | 24% |
| Rural^c^ | 157 | 13% | 80 | 15% | 8% | 8% |

*^a^ Note differing time periods and not an exact match on time period compared with the study cohort (2006-2013), these were the most timely publicly available data from the Cancer Institute NSW website.*

*^b^ For each cancer site, there are an additional 6% aged <45 included in the NSW statistics.*

*^c^ Includes outer regional, remote and very remote areas.*

*Table C. Summary statistics for excess costs, by phase of care and by year/month for colon and rectal cancer cases diagnosed 2006-2013****.***

|  | **Colon cancer** | | | | | **Rectal cancer** | | | |
| --- | --- | --- | --- | --- | --- | --- | --- | --- | --- |
|  | **No. of cases** | **Mean (SD)** | **Median** | **Quartiles 1 - 3** | **No. of cases** | | **Mean (SD)** | **Median** | **Quartiles 1 - 3** |
| Phase of care |  |  |  |  |  | |  |  |  |
| Initial | 1012 | $50,434 ($41,971) | $39,912 | $25,448 - $61,574 | 484 | | $60,877 ($46,086) | $55,852 | $31,801 - $82,246 |
| Continuing (per year) | 905 | $6,779 ($26,044) | $967 | -$4,326 - $9,350 | 443 | | $8,336 ($26,439) | $1,457 | -$3,124 - $11,050 |
| Terminal | 445 | $74,952 ($64,677) | $56,828 | $26,840 - $105,711 | 181 | | $67,733 ($54,338) | $57,624 | $27,069 - $100,006 |
| Annual costs around diagnosis |  |  |  |  |  | |  |  |  |
| >1-2 years before diagnosis | 1200 | $3,338 ($18,193) | $246 | -$4,007 - $6,714 | 546 | | $2,453 ($20,380) | -$401 | -$3,774 - $4,167 |
| 0-1 year after diagnosis | 1200 | $55,951 ($47,844) | $42,900 | $25,949 - $70,822 | 546 | | $62,971 ($46,470) | $56,966 | $33,269 - $87,072 |
| >1-2 years after diagnosis | 1011 | $11,964 ($35,062) | $2,107 | -$3,770 - $13,229 | 484 | | $12,416 ($34,493) | $2,644 | -$1,914 - $15,375 |
| >2-3 years after diagnosis | 830 | $9,613 ($31,424) | $308 | -$4,192 - $10,638 | 404 | | $11,891 ($36,535) | $1,152 | -$3,243 - $13,304 |
| >3-4 years after diagnosis | 632 | $9,677 ($32,206) | $367 | -$4,782 - $10,997 | 318 | | $10,136 ($30,341) | $989 | -$3,111 - $12,693 |
| >4-5 years after diagnosis | 451 | $7,237 ($32,758) | $121 | -$4,953 - $6,803 | 229 | | $8,187 ($30,723) | $237 | -$4,851 - $10,789 |
| By month relative to diagnosis |  |  |  |  |  | |  |  |  |
| Month 3 before diagnosis | 1200 | $119 ($3,155) | -$64 | -$333 - $192 | 546 | | $0 ($2,704) | -$59 | -$267 - $155 |
| Month 2 before diagnosis | 1200 | $429 ($3,300) | -$10 | -$261 - $349 | 546 | | $293 ($2,684) | -$17 | -$217 - $253 |
| Month 1 before diagnosis | 1200 | $2,113 ($5,910) | $320 | -$78 - $2,370 | 546 | | $1,133 ($5,822) | $118 | -$136 - $846 |
| Month 1 after diagnosis | 1200 | $26,239 ($19,633) | $25,169 | $8,681 - $37,563 | 546 | | $16,728 ($18,770) | $7,758 | $3,798 - $30,963 |
| Month 2 after diagnosis | 1163 | $9,564 ($15,789) | $1,930 | $38 - $14,755 | 535 | | $11,470 ($18,101) | $4,356 | $447 - $19,855 |
| Month 3 after diagnosis | 1139 | $4,237 ($9,439) | $447 | -$106 - $4,335 | 527 | | $6,347 ($11,843) | $1,984 | $82 - $6,837 |
| Month 4 after diagnosis | 1121 | $3,216 ($9,237) | $300 | -$125 - $2,892 | 515 | | $5,918 ($14,018) | $1,031 | $37 - $6,325 |
| Month 5 after diagnosis | 1104 | $2,929 ($7,964) | $269 | -$112 - $2,575 | 514 | | $6,158 ($11,740) | $802 | -$15 - $8,295 |
| Month 6 after diagnosis | 1086 | $2,422 ($6,447) | $204 | -$152 - $2,081 | 511 | | $4,266 ($9,974) | $603 | -$30 - $3,550 |
| By month relative to death |  |  |  |  |  | |  |  |  |
| Month 6 before death | 344 | $6,367 ($12,099) | $1,274 | -$33 - $8,347 | 151 | | $4,511 ($8,288) | $1,044 | $68 - $6,692 |
| Month 5 before death | 358 | $6,155 ($11,181) | $1,164 | -$2 - $8,744 | 152 | | $5,059 ($8,837) | $1,469 | $301 - $6,446 |
| Month 4 before death | 373 | $8,001 ($13,384) | $2,172 | $116 - $10,851 | 152 | | $5,936 ($18,511) | $1,519 | $68 - $5,848 |
| Month 3 before death | 389 | $8,111 ($12,988) | $2,229 | $170 - $12,290 | 164 | | $7,458 ($13,232) | $2,380 | $250 - $8,953 |
| Month 2 before death | 410 | $9,075 ($13,901) | $3,098 | $268 - $12,440 | 170 | | $7,793 ($12,020) | $2,508 | $165 - $12,231 |
| Final month of life | 445 | $20,670 ($25,592) | $14,700 | $5,550 - $28,440 | 181 | | $20,088 ($21,490) | $14,707 | $6,049 - $29,648 |

*SD: standard deviation. Costs reported in Australian dollars for the year 2020, for cases alive at the start of each time period.*

*Table D. Summary statistics for excess hospital days, by phase of care and by year/month for colon and rectal cancer cases diagnosed 2006-2013****.***

|  | **Colon cancer** | | | | **Rectal cancer** | | | |
| --- | --- | --- | --- | --- | --- | --- | --- | --- |
|  | **No. of cases** | **Mean (SD)** | **Median** | **Quartiles 1 - 3** | **No. of cases** | **Mean (SD)** | **Median** | **Quartiles 1 - 3** |
| Phase of care |  |  |  |  |  |  |  |  |
| Initial | 1012 | 16.0 (21.2) | 10 | 6 - 20 | 484 | 18.5 (20.1) | 14 | 7 - 25 |
| Continuing (per year) | 905 | 1.7 (14.6) | 0 | -2 - 2 | 443 | 2.0 (10.7) | 0.1 | -1 - 2 |
| Terminal | 445 | 34.1 (33.0) | 25 | 11 - 49 | 181 | 30.4 (34.1) | 22 | 8 - 40 |
| Annual days relative to diagnosis |  |  |  |  |  |  |  |  |
| >0-1 year before diagnosis | 1200 | 1.1 (12.5) | 0 | -1 - 2 | 546 | 0.7 (13.8) | 0 | -1 - 1 |
| 0-1 year after diagnosis | 1200 | 19.6 (24.9) | 11 | 6 - 25 | 546 | 20.4 (22.8) | 15 | 7 - 28 |
| >1-2 years after diagnosis | 1011 | 4.3 (17.5) | 0.5 | -1 - 4 | 484 | 3.8 (14.2) | 0.7 | -1 - 4 |
| >2-3 years after diagnosis | 830 | 3.2 (15.4) | 0 | -1 - 3 | 404 | 4.1 (16.4) | 0 | -1 - 3 |
| >3-4 years after diagnosis | 632 | 3.4 (16.7) | 0 | -2 - 2 | 318 | 3.4 (16.3) | 0 | -1 - 2 |
| >4-5 years after diagnosis | 451 | 3.5 (21.6) | 0 | -1 - 1 | 229 | 3.3 (16.6) | 0 | -1 - 2 |
| Monthly days around diagnosis |  |  |  |  |  |  |  |  |
| Month 3 before diagnosis | 1200 | 0.0 (1.8) | 0 | 0 - 0 | 546 | 0.0 (1.9) | 0 | 0 - 0 |
| Month 2 before diagnosis | 1200 | 0.1 (2.2) | 0 | 0 - 0 | 546 | 0.1 (2.3) | 0 | 0 - 0 |
| Month 1 before diagnosis | 1200 | 0.8 (3.2) | 0 | 0 - 1 | 546 | 0.4 (3.0) | 0 | 0 - 0 |
| Month 1 after diagnosis | 1200 | 8.9 (7.9) | 7 | 2 - 12 | 546 | 5.4 (7.5) | 2 | 1 - 8 |
| Month 2 after diagnosis | 1163 | 3.8 (6.8) | 0 | 0 - 6 | 535 | 3.7 (6.9) | 0 | 0 - 6 |
| Month 3 after diagnosis | 1139 | 1.6 (5.2) | 0 | 0 - 0 | 527 | 2.1 (5.6) | 0 | 0 - 1 |
| Month 4 after diagnosis | 1121 | 1.1 (4.3) | 0 | 0 - 0 | 515 | 1.9 (4.9) | 0 | 0 - 1 |
| Month 5 after diagnosis | 1104 | 0.9 (3.8) | 0 | 0 - 0 | 514 | 2.1 (5.1) | 0 | 0 - 2 |
| Month 6 after diagnosis | 1086 | 0.6 (3.1) | 0 | 0 - 0 | 511 | 1.5 (4.6) | 0 | 0 - 0 |
| Monthly days at the end of life |  |  |  |  |  |  |  |  |
| Month 6 before death | 344 | 2.5 (5.9) | 0 | 0 - 2 | 151 | 1.4 (5.2) | 0 | 0 - 0 |
| Month 5 before death | 358 | 2.6 (6.3) | 0 | 0 - 2 | 152 | 1.5 (5.0) | 0 | 0 - 1 |
| Month 4 before death | 373 | 3.5 (7.1) | 0 | 0 - 4 | 152 | 2.6 (7.4) | 0 | 0 - 1 |
| Month 3 before death | 389 | 4.1 (7.8) | 0 | 0 - 6 | 164 | 4.2 (8.1) | 0 | 0 - 5 |
| Month 2 before death | 410 | 5.0 (8.1) | 1 | 0 - 7 | 170 | 5.6 (9.8) | 0 | 0 - 10 |
| Final month of life | 445 | 11.4 (9.9) | 9 | 2 - 19 | 181 | 10.7 (10.6) | 8 | 2 - 18 |

*SD: standard deviation. Excess hospital days are for cases alive at the start of each time period.*

*Table E. Anti-cancer treatments received by patients’ characteristics, for colon and rectal cancer cases diagnosed 2006-2013****.***

|  | **Colon cancer** | | | | | **Rectal cancer** | | | | |
| --- | --- | --- | --- | --- | --- | --- | --- | --- | --- | --- |
| **Characteristic** | **No. of cases** | **% had any treatment** | **% had surgery** | **% had chemotherapy** | **% had radiotherapy** | **No. of cases** | **% had any treatment** | **% had surgery** | **% had chemotherapy** | **% had radiotherapy** |
| **Stage at diagnosis** |  |  |  |  |  |  |  |  |  |  |
| Localised | 398 | 89% | 89% | 10% | 4% | 187 | 86% | 79% | 31% | 32% |
| Regional | 492 | 97% | 95% | 54% | 7% | 221 | 97% | 91% | 72% | 45% |
| Distant metastases | 243 | 80% | 63% | 62% | 14% | 90 | 86% | 58% | 74% | 43% |
| Unknown | 67 | 69% | 60% | 16% | 8% | 48 | 73% | 63% | 35% | 29% |
| **Sex** |  |  |  |  |  |  |  |  |  |  |
| Female | 576 | 88% | 83% | 38% | 9% | 355 | 88% | 78% | 55% | 39% |
| Male | 624 | 91% | 86% | 40% | 7% | 191 | 92% | 79% | 56% | 38% |
| **Age at diagnosis** |  |  |  |  |  |  |  |  |  |  |
| 45-54 | 62 | 95% | 89% | 50% | <10% | 58 | 95% | 90% | 72% | 41% |
| 55-64 | 220 | 93% | 86% | 57% | 10% | 147 | 93% | 80% | 65% | 50% |
| 65-74 | 417 | 92% | 86% | 45% | 8% | 183 | 91% | 84% | 55% | 36% |
| 75+ | 501 | 85% | 82% | 25% | 6% | 158 | 82% | 67% | 40% | 32% |
| **Year of diagnosis** |  |  |  |  |  |  |  |  |  |  |
| 2006-2008 | 165 | 90% | 87% | 43% | 10% | 93 | 90% | 83% | 57% | 27% |
| 2009 | 204 | 89% | 81% | 39% | 8% | 90 | 87% | 77% | 50% | 38% |
| 2010 | 219 | 90% | 85% | 46% | 7% | 103 | 88% | 78% | 54% | 44% |
| 2011 | 216 | 92% | 88% | 38% | 8% | 97 | 88% | 70% | 53% | 40% |
| 2012 | 187 | 88% | 83% | 36% | 4% | 73 | 93% | 85% | 62% | 44% |
| 2013 | 209 | 89% | 84% | 34% | 7% | 90 | 90% | 81% | 58% | 42% |
| **Smoking status** |  |  |  |  |  |  |  |  |  |  |
| Never smoker | 649 | 91% | 86% | 37% | 7% | 251 | 90% | 81% | 55% | 42% |
| Ex-smoker quit >15yrs | 325 | 90% | 85% | 38% | 8% | 170 | 87% | 76% | 54% | 37% |
| Ex-smoker quit ≤15yrs | 136 | 85% | 80% | 46% | 10% | 81 | 88% | 79% | 48% | 35% |
| Current smoker | 90 | 88% | 78% | 47% | 6% | 44 | 96% | 77% | 73% | 39% |
| **Remoteness of residence** |  |  |  |  |  |  |  |  |  |  |
| Major cities | 630 | 89% | 84% | 39% | 7% | 256 | 90% | 82% | 55% | 36% |
| Inner regional | 413 | 91% | 86% | 39% | 7% | 210 | 88% | 74% | 54% | 42% |
| Outer regional/(Very) Remote | 157 | 89% | 83% | 40% | 11% | 80 | 91% | 79% | 61% | 40% |
| **Socioeconomic quintile** |  |  |  |  |  |  |  |  |  |  |
| Most disadvantaged | 298 | 87% | 82% | 40% | 8% | 126 | 90% | 72% | 52% | 37% |
| Quintile 2 | 257 | 89% | 85% | 39% | 9% | 122 | 86% | 79% | 58% | 40% |
| Quintile 3 | 222 | 91% | 85% | 37% | 6% | 109 | 90% | 83% | 55% | 39% |
| Quintile 4 | 181 | 91% | 85% | 37% | 7% | 94 | 93% | 80% | 55% | 42% |
| Least disadvantaged | 216 | 91% | 86% | 41% | 8% | 85 | 87% | 82% | 54% | 37% |
| Missing | 26 | 92% | 89% | 54% | <10% | 10 | 100% | 70% | 80% | 40% |
| **Health insurance status** |  |  |  |  |  |  |  |  |  |  |
| Private insurance | 691 | 92% | 87% | 43% | 8% | 315 | 91% | 84% | 56% | 38% |
| Concession card | 322 | 84% | 79% | 31% | 7% | 129 | 88% | 74% | 50% | 36% |
| None | 155 | 90% | 85% | 41% | 7% | 86 | 86% | 69% | 58% | 44% |
| Missing | 32 | 91% | 81% | 25% | <10% | 16 | 94% | 69% | 69% | 56% |
| **Comorbidity score** |  |  |  |  |  |  |  |  |  |  |
| 0 | 990 | 91% | 86% | 42% | 8% | 474 | 91% | 80% | 58% | 41% |
| 1 | 116 | 91% | 85% | 28% | 9% | 42 | 91% | 81% | 45% | 33% |
| 2+ | 94 | 76% | 72% | 21% | <5% | 30 | 76% | 50% | 33% | 20% |
| **BMI** |  |  |  |  |  |  |  |  |  |  |
| Normal/Underweight | 422 | 89% | 85% | 38% | 6% | 181 | 87% | 74% | 55% | 38% |
| Overweight | 436 | 92% | 87% | 40% | 7% | 209 | 90% | 81% | 57% | 39% |
| Obese | 259 | 87% | 81% | 39% | 9% | 126 | 92% | 83% | 54% | 42% |
| Missing | 83 | 89% | 83% | 41% | 13% | 30 | 90% | 73% | 47% | 33% |
| **Self-reported CRC screening** |  |  |  |  |  |  |  |  |  |  |
| Yes | 542 | 91% | 87% | 37% | 8% | 160 | 88% | 82% | 46% | 33% |
| No | 637 | 88% | 83% | 41% | 7% | 376 | 90% | 77% | 60% | 42% |
| Missing | 21 | 76% | 67% | 29% | 0% | 10 | 80% | 80% | 40% | 30% |
| **Self-reported FOBT** |  |  |  |  |  |  |  |  |  |  |
| Yes | 289 | 92% | 87% | 39% | 9% | 102 | 87% | 83% | 44% | 30% |
| No | 911 | 89% | 84% | 39% | 7% | 444 | 90% | 77% | 58% | 41% |

*Note: Using codes/item numbers in Table A to identify colorectal cancer treatment. Records included were from 30 days prior to cancer registry diagnosis date onwards – this is not restricted to a single phase of care.*

*Table F.* *Relative effects and corresponding p-values from multivariable regression for excess healthcare costs for colon and rectal cancer cases diagnosed 2006-2013 in the 45 and Up Study, separately for each phase of care.*

|  | **Initial phase** | | **Continuing phase** | | **Terminal phase** | |
| --- | --- | --- | --- | --- | --- | --- |
| **Category** | **Colon** | **Rectal** | **Colon** | **Rectal** | **Colon** | **Rectal** |
| No. of cases included | 944 | 462 | 844 | 420 | 424 | 169 |
| Stage at diagnosis | *p<0.0001* | *p<0.0001* | *p<0.0001* | *p<0.0001* | *p=0.004* | *p=0.45* |
| Localised | (reference) | (reference) | (reference) | (reference) | (reference) | (reference) |
| Regional | 1.23 (1.18-1.29) | 1.23 (1.15-1.32) | 1.08 (1.03-1.13) | 1.10 (1.03-1.18) | 1.01 (0.88-1.15) | 0.98 (0.82-1.16) |
| Distant metastases | 1.53 (1.42-1.64) | 1.38 (1.25-1.54) | 1.32 (1.21-1.43) | 1.70 (1.52-1.91) | 1.03 (0.91-1.18) | 1.03 (0.87-1.22) |
| Unknown | 0.95 (0.87-1.04) | 0.87 (0.78-0.97) | 1.01 (0.93-1.10) | 0.97 (0.88-1.07) | 0.74 (0.60-0.90) | 0.86 (0.67-1.10) |
| Sex | *p=0.94* | *p=0.63* | *p=0.38* | *p=0.10* | *p=0.31* | *p=0.19* |
| Female | 1.00 (0.96-1.05) | 1.02 (0.95-1.09) | 1.02 (0.98-1.06) | 1.05 (0.99-1.12) | 0.96 (0.88-1.04) | 0.92 (0.80-1.04) |
| Male | (reference) | (reference) | (reference) | (reference) | (reference) | (reference) |
| Age at diagnosis (years) | *p<0.0001* | *p=0.04* | *p<0.0001* | *p=0.49* | *p<0.0001* | *p=0.001* |
| 45-54 | 1.10 (1.00-1.20) | 1.15 (1.03-1.28) | 1.00 (0.91-1.09) | 1.05 (0.95-1.16) | 1.15 (0.88-1.50) | 0.97 (0.72-1.30) |
| 55-64 | 1.03 (0.97-1.10) | 1.09 (1.00-1.18) | 1.01 (0.96-1.07) | 1.02 (0.95-1.10) | 1.10 (0.96-1.26) | 1.14 (0.95-1.36) |
| 65-74 | (reference) | (reference) | (reference) | (reference) | (reference) | (reference) |
| 75+ | 0.90 (0.86-0.95) | 1.03 (0.95-1.12) | 0.87 (0.83-0.91) | 0.97 (0.90-1.05) | 0.72 (0.66-0.80) | 0.82 (0.71-0.95) |
| Year of diagnosis | *p=0.49* | *p=0.46* | *p=0.50* | *p=0.40* | *p=0.08* | *p=0.44* |
| 2006-2008 | 0.97 (0.90-1.04) | 1.00 (0.90-1.11) | 1.05 (0.98-1.13) | 0.98 (0.89-1.08) | 0.96 (0.83-1.10) | 0.99 (0.82-1.20) |
| 2009 | 1.00 (0.94-1.08) | 0.99 (0.89-1.10) | 1.05 (0.98-1.12) | 1.01 (0.91-1.12) | 1.00 (0.88-1.14) | 1.11 (0.90-1.36) |
| 2010 | (reference) | (reference) | (reference) | (reference) | (reference) | (reference) |
| 2011 | 0.94 (0.88-1.01) | 0.98 (0.88-1.08) | 1.03 (0.97-1.11) | 1.03 (0.94-1.14) | 1.05 (0.91-1.20) | 0.93 (0.77-1.13) |
| 2012 | 0.97 (0.90-1.04) | 1.00 (0.89-1.11) | 1.06 (0.99-1.14) | 1.07 (0.96-1.18) | 0.93 (0.80-1.08) | 1.13 (0.90-1.41) |
| 2013 | 0.97 (0.91-1.04) | 1.08 (0.98-1.20) | 1.01 (0.94-1.08) | 0.95 (0.87-1.05) | 1.17 (1.01-1.35) | 1.01 (0.80-1.28) |
| Smoking status | *p=0.03* | *p=0.05* | *p=0.03* | *p=0.14* | *p=0.64* | *p=0.21* |
| Never smoker | (reference) | (reference) | (reference) | (reference) | (reference) | (reference) |
| Ex-smoker quit >15 years | 0.99 (0.94-1.04) | 0.92 (0.86-0.99) | 1.02 (0.97-1.07) | 1.04 (0.97-1.12) | 1.06 (0.96-1.16) | 1.10 (0.95-1.27) |
| Ex-smoker quit ≤15 years | 1.01 (0.94-1.08) | 0.98 (0.89-1.08) | 1.00 (0.94-1.08) | 0.94 (0.85-1.03) | 1.01 (0.88-1.16) | 1.11 (0.92-1.33) |
| Current smoker | 0.88 (0.81-0.96) | 1.06 (0.95-1.19) | 0.89 (0.82-0.97) | 0.97 (0.86-1.09) | 0.97 (0.83-1.13) | 0.87 (0.68-1.12) |
| Remoteness of place of residence | *p=0.02* | *p=0.92* | *p=0.63* | *p=0.80* | *p=0.002* | *p=0.93* |
| Major cities | (reference) | (reference) | (reference) | (reference) | (reference) | (reference) |
| Inner regional | 0.94 (0.89-0.98) | 0.99 (0.92-1.06) | 1.01 (0.96-1.06) | 0.98 (0.92-1.05) | 0.91 (0.83-1.00) | 0.98 (0.85-1.12) |
| Outer regional/Remote/Very remote | 0.93 (0.87-1.00) | 1.00 (0.91-1.10) | 1.03 (0.97-1.10) | 0.97 (0.89-1.07) | 0.79 (0.69-0.90) | 0.97 (0.81-1.16) |
| Socioeconomic quintile | *p=0.42* | *p=0.40* | *p=0.03* | *p=0.10* | *p=0.81* | *p=0.25* |
| Most disadvantaged quintile | 1.00 (0.93-1.06) | 1.01 (0.92-1.11) | 1.00 (0.94-1.07) | 0.94 (0.86-1.03) | 0.99 (0.87-1.12) | 0.88 (0.74-1.05) |
| Quintile 2 | 0.98 (0.92-1.04) | 1.09 (0.99-1.20) | 1.00 (0.94-1.07) | 0.93 (0.85-1.02) | 0.99 (0.88-1.12) | 0.88 (0.73-1.06) |
| Quintile 3 | (reference) | (reference) | (reference) | (reference) | (reference) | (reference) |
| Quintile 4 | 0.99 (0.92-1.06) | 1.04 (0.94-1.15) | 0.98 (0.92-1.05) | 0.88 (0.80-0.96) | 1.02 (0.88-1.18) | 0.95 (0.78-1.16) |
| Least disadvantaged quintile | 0.94 (0.88-1.01) | 1.06 (0.96-1.17) | 1.09 (1.02-1.17) | 0.92 (0.84-1.01) | 1.07 (0.93-1.24) | 1.09 (0.84-1.41) |
| Health insurance status | *p=0.01* | *p<0.0001* | *p=0.31* | *p=0.63* | *p=0.002* | *p=0.35* |
| Private insurance | (reference) | (reference) | (reference) | (reference) | (reference) | (reference) |
| Concession card | 0.96 (0.91-1.01) | 0.90 (0.83-0.97) | 1.02 (0.97-1.08) | 0.96 (0.89-1.04) | 0.83 (0.75-0.92) | 0.93 (0.82-1.07) |
| None | 0.91 (0.85-0.97) | 0.82 (0.75-0.90) | 0.97 (0.91-1.03) | 1.00 (0.92-1.09) | 0.90 (0.79-1.03) | 0.90 (0.76-1.06) |
| Comorbidity score | *p<0.0001* | *p=0.001* | *p=0.01* | *p=0.22* | *p=0.75* | *p=0.12* |
| 0 | (reference) | (reference) | (reference) | (reference) | (reference) | (reference) |
| 1 | 1.11 (1.02-1.20) | 1.14 (1.01-1.28) | 1.10 (1.01-1.19) | 1.10 (0.98-1.24) | 1.04 (0.92-1.18) | 1.14 (0.92-1.41) |
| 2+ | 1.21 (1.10-1.32) | 1.43 (1.13-1.80) | 1.11 (1.02-1.22) | 1.08 (0.86-1.34) | 1.03 (0.91-1.16) | 0.88 (0.73-1.04) |
| Self-reported CRC screening | *p=0.04* |  |  |  |  |  |
| No | (reference) |  |  |  |  |  |
| Yes | 1.04 (1.00-1.09) |  |  |  |  |  |
| Self-reported FOBT |  |  |  |  | *p=0.04* |  |
| No |  |  |  |  | (reference) |  |
| Yes |  |  |  |  | 1.11 (1.00-1.23) |  |

** The relative effects are the exponentiated beta estimates and confidence limits from the log-linked gamma regression, which were calculated including an offset of the excess cost values by +$50,000.*

*Notes: Excludes cases with missing values for any of the listed variables and any costs that were outliers. Relative effects are adjusted for all listed variables from stage at diagnosis to comorbidity score. Regressions were tested for the separate inclusion of self-reported CRC screening, self-reported FOBT and BMI – these were included if the p-value was <0.10, which applied for 2/6 regressions above. BMI had p>0.10 in all regressions so it was not included in the final analyses. The regression analysis yielded similar results when unknown BMI was included as a separate category.*

*Table G. Relative effects and corresponding p-values from multivariable regression for excess days in hospital for colon and rectal cancer cases diagnosed 2006-2013 in the 45 and Up Study, separately for each phase of care.*

|  | **Initial phase** | | **Continuing phase** | | **Terminal phase** | |
| --- | --- | --- | --- | --- | --- | --- |
| **Category** | **Colon** | **Rectal** | **Colon** | **Rectal** | **Colon** | **Rectal** |
| No. of cases included | 934 | 438 | 841 | 389 | 426 | 162 |
| Stage at diagnosis | *p<0.0001* | *p<0.0001* | *p=0.10* | *p=0.0003* | *p=0.09* | *p=0.47* |
| Localised | (reference) | (reference) | (reference) | (reference) | (reference) | (reference) |
| Regional | 1.11 (1.08-1.15) | 1.08 (1.03-1.13) | 1.02 (1.00-1.04) | 1.01 (0.98-1.03) | 1.01 (0.91-1.12) | 0.94 (0.80-1.10) |
| Distant metastases | 1.20 (1.15-1.26) | 1.09 (1.02-1.18) | 1.04 (1.00-1.08) | 1.08 (1.04-1.13) | 0.96 (0.87-1.07) | 1.00 (0.86-1.17) |
| Unknown | 0.96 (0.91-1.02) | 0.92 (0.86-0.98) | 1.01 (0.97-1.05) | 0.99 (0.96-1.02) | 0.84 (0.71-0.99) | 0.87 (0.69-1.10) |
| Sex | *p=0.49* | *p=0.30* | *p=0.22* | *p=0.10* | *p=0.41* | *p=0.28* |
| Female | 1.01 (0.98-1.04) | 1.02 (0.98-1.07) | 1.01 (0.99-1.03) | 1.02 (1.00-1.04) | 1.03 (0.96-1.11) | 1.07 (0.95-1.20) |
| Male | (reference) | (reference) | (reference) | (reference) | (reference) | (reference) |
| Age at diagnosis (years) | *p=0.14* | *p=0.14* | *p<0.0001* | *p=0.08* | *p=0.10* | *p=0.43* |
| 45-54 | 1.04 (0.98-1.11) | 1.03 (0.96-1.11) | 0.99 (0.95-1.03) | 1.03 (1.00-1.07) | 1.05 (0.85-1.31) | 0.92 (0.72-1.19) |
| 55-64 | 0.99 (0.95-1.03) | 1.03 (0.97-1.08) | 1.00 (0.97-1.02) | 1.00 (0.98-1.03) | 0.96 (0.85-1.07) | 1.10 (0.94-1.29) |
| 65-74 | (reference) | (reference) | (reference) | (reference) | (reference) | (reference) |
| 75+ | 0.97 (0.94-1.01) | 1.07 (1.01-1.13) | 0.94 (0.92-0.96) | 0.98 (0.96-1.01) | 0.91 (0.84-0.99) | 1.01 (0.89-1.16) |
| Year of diagnosis | *p=0.42* | *p=0.80* | *p=0.59* | *p=0.60* | *p=0.17* | *p=0.15* |
| 2006-2008 | 0.99 (0.94-1.04) | 1.00 (0.94-1.08) | 0.99 (0.96-1.02) | 1.00 (0.97-1.04) | 0.97 (0.87-1.09) | 0.84 (0.71-1.00) |
| 2009 | 1.03 (0.98-1.08) | 0.98 (0.91-1.05) | 1.00 (0.97-1.03) | 1.01 (0.98-1.05) | 1.00 (0.91-1.12) | 0.95 (0.79-1.14) |
| 2010 | (reference) | (reference) | (reference) | (reference) | (reference) | (reference) |
| 2011 | 0.98 (0.94-1.03) | 1.00 (0.93-1.07) | 0.98 (0.95-1.01) | 1.01 (0.98-1.05) | 1.07 (0.96-1.20) | 0.87 (0.73-1.04) |
| 2012 | 0.99 (0.94-1.04) | 1.00 (0.93-1.07) | 0.99 (0.95-1.02) | 1.03 (0.99-1.06) | 0.98 (0.87-1.10) | 1.02 (0.83-1.25) |
| 2013 | 1.01 (0.96-1.06) | 1.03 (0.97-1.11) | 0.98 (0.95-1.01) | 1.00 (0.97-1.03) | 1.12 (1.00-1.26) | 0.83 (0.68-1.03) |
| Smoking status | *p=0.31* | *p=0.01* | *p=0.31* | *p=0.02* | *p=0.58* | *p=0.40* |
| Never smoker | (reference) | (reference) | (reference) | (reference) | (reference) | (reference) |
| Ex-smoker quit >15 years | 0.98 (0.95-1.02) | 0.96 (0.91-1.01) | 1.00 (0.98-1.02) | 1.01 (0.98-1.03) | 1.06 (0.97-1.14) | 1.08 (0.95-1.23) |
| Ex-smoker quit ≤15 years | 1.00 (0.95-1.05) | 1.01 (0.95-1.08) | 1.01 (0.98-1.04) | 0.96 (0.93-0.99) | 1.02 (0.91-1.15) | 1.08 (0.91-1.27) |
| Current smoker | 0.95 (0.90-1.01) | 1.10 (1.02-1.19) | 0.97 (0.93-1.00) | 0.97 (0.94-1.01) | 0.99 (0.88-1.12) | 0.92 (0.74-1.15) |
| Remoteness of place of residence | *p=0.03* | *p=0.89* | *p=0.83* | *p=0.30* | *p=0.15* | *p=0.50* |
| Major cities | (reference) | (reference) | (reference) | (reference) | (reference) | (reference) |
| Inner regional | 0.96 (0.93-0.99) | 1.00 (0.96-1.05) | 1.00 (0.98-1.02) | 0.99 (0.97-1.01) | 0.95 (0.88-1.03) | 0.95 (0.84-1.07) |
| Outer regional/Remote/Very remote | 0.97 (0.92-1.01) | 1.02 (0.95-1.08) | 1.01 (0.98-1.04) | 0.98 (0.95-1.01) | 0.90 (0.81-1.01) | 0.91 (0.78-1.07) |
| Socioeconomic quintile | *p=0.82* | *p=0.14* | *p=0.03* | *p=0.25* | *p=0.23* | *p=0.22* |
| Most disadvantaged quintile | 1.00 (0.95-1.04) | 1.02 (0.95-1.08) | 0.99 (0.96-1.02) | 0.99 (0.96-1.02) | 0.97 (0.88-1.08) | 1.02 (0.87-1.19) |
| Quintile 2 | 0.98 (0.93-1.02) | 1.08 (1.01-1.15) | 0.99 (0.96-1.02) | 1.00 (0.97-1.03) | 0.97 (0.87-1.07) | 0.95 (0.81-1.12) |
| Quintile 3 | (reference) | (reference) | (reference) | (reference) | (reference) | (reference) |
| Quintile 4 | 0.99 (0.95-1.04) | 1.06 (0.99-1.13) | 0.98 (0.95-1.01) | 0.97 (0.94-1.00) | 0.96 (0.85-1.08) | 0.90 (0.75-1.07) |
| Least disadvantaged quintile | 0.98 (0.94-1.03) | 1.06 (0.99-1.13) | 1.03 (1.00-1.06) | 0.99 (0.96-1.02) | 1.09 (0.97-1.23) | 1.14 (0.91-1.43) |
| Health insurance status | *p=0.29* | *p=0.01* | *p=0.90* | *p=0.35* | *p=0.05* | *p=0.86* |
| Private insurance | (reference) | (reference) | (reference) | (reference) | (reference) | (reference) |
| Concession card | 0.98 (0.95-1.02) | 1.00 (0.95-1.06) | 0.99 (0.97-1.02) | 0.99 (0.96-1.01) | 0.91 (0.84-0.98) | 0.97 (0.86-1.09) |
| None | 0.97 (0.92-1.01) | 0.92 (0.86-0.97) | 1.00 (0.97-1.03) | 1.01 (0.98-1.04) | 0.93 (0.84-1.04) | 1.00 (0.86-1.15) |
| Comorbidity score | *p<0.0001* | *p<0.0001* | *p<0.0001* | *p=0.001* | *p=0.15* | *p=0.47* |
| 0 | (reference) | (reference) | (reference) | (reference) | (reference) | (reference) |
| 1 | 1.15 (1.09-1.21) | 1.05 (0.97-1.14) | 1.06 (1.03-1.10) | 1.07 (1.03-1.11) | 1.09 (0.99-1.21) | 1.10 (0.90-1.34) |
| 2+ | 1.20 (1.13-1.28) | 1.46 (1.25-1.70) | 1.08 (1.03-1.12) | 1.09 (1.01-1.17) | 1.06 (0.96-1.17) | 0.95 (0.81-1.12) |
| Body mass index (kg/m2) |  |  |  |  |  | *p=0.04* |
| Normal/Underweight |  |  |  |  |  | (reference) |
| Overweight |  |  |  |  |  | 1.08 (0.96-1.22) |
| Obese |  |  |  |  |  | 0.89 (0.77-1.03) |
| Self-reported CRC screening | *p=0.04* |  |  |  |  |  |
| No | (reference) |  |  |  |  |  |
| Yes | 1.03 (1.00-1.06) |  |  |  |  |  |

** The relative effects are the exponentiated beta estimates and confidence limits from the gamma log-linked regression, which was calculated including an offset of the excess hospital day values by +50.*

*Notes: Excludes cases with missing values for any of the listed variables and any costs that were outliers. Relative effects are adjusted for all listed variables from stage at diagnosis to comorbidity score. Regressions were tested for the separate inclusion of self-reported CRC screening, self-reported FOBT and BMI – these were included if the p-value was <0.10, which applied for 2/6 regressions above. Self-reported FOBT had p>0.10 in all regressions so it was not included in the final models. The regression analysis yielded similar results when unknown BMI was included as a separate category.*

*Table H. Excess costs for colon and rectal cases diagnosed 2006-2013, by source of costs, for selected time periods.*

|  | **Colon cancer (n=1200)** | | | | | **Rectal cancer (n=546)** | | | | |
| --- | --- | --- | --- | --- | --- | --- | --- | --- | --- | --- |
| **Time period** | **No. of cases** | **Mean excess cost per case** | **Hospital-**  **based (%)** | **MBS**  **(%)** | **PBS**  **(%)** | **No. of cases** | **Mean excess cost per case** | **Hospital-**  **based (%)** | **MBS**  **(%)** | **PBS**  **(%)** |
| Phase of care |  |  |  |  |  |  |  |  |  |  |
| Initial | 1012 | $50,434 | 79% | 12% | 9% | 484 | $60,877 | 75% | 16% | 8% |
| Continuing (per year) | 905 | $6,779 | 50% | 20% | 30% | 443 | $8,336 | 45% | 20% | 35% |
| Terminal | 445 | $74,952 | 77% | 8% | 14% | 181 | $67,733 | 73% | 9% | 17% |
| Annual costs around diagnosis | | |  |  |  |  |  |  |  |  |
| >1-2 years before diagnosis | 1178 | -$111 | N/A | N/A | N/A | 536 | $481 | 42% | 12% | 47% |
| >0-1 year before diagnosis | 1200 | $3,338 | 74% | 26% | 0% | 546 | $2,453 | 68% | 22% | 10% |
| 0-1 year after diagnosis | 1200 | $55,951 | 79% | 11% | 10% | 546 | $62,971 | 76% | 15% | 9% |
| >1-2 years after diagnosis | 1011 | $11,964 | 63% | 17% | 20% | 484 | $12,416 | 56% | 17% | 27% |
| >2-3 years after diagnosis | 830 | $9,613 | 68% | 15% | 17% | 404 | $11,891 | 62% | 15% | 23% |
| >3-4 years after diagnosis | 632 | $9,677 | 66% | 14% | 20% | 318 | $10,136 | 64% | 16% | 21% |
| >4-5 years after diagnosis | 451 | $7,237 | 71% | 12% | 17% | 229 | $8,187 | 61% | 16% | 23% |
| Monthly costs around diagnosis | | |  |  |  |  |  |  |  |  |
| Month 3 before diagnosis | 1200 | $119 | 58% | 34% | 8% | 546 | $0 | N/A | N/A | N/A |
| Month 2 before diagnosis | 1200 | $429 | *64%* | *36%* | *0%* | 546 | $293 | 67% | 31% | 2% |
| Month 1 before diagnosis | 1200 | $2,113 | 76% | 23% | 0% | 546 | $1,133 | 63% | 35% | 2% |
| Month 1 after diagnosis | 1200 | $26,239 | 90% | 10% | 0% | 546 | $16,728 | 83% | 16% | 0% |
| Month 2 after diagnosis | 1163 | $9,564 | 90% | 7% | 4% | 535 | $11,470 | 83% | 16% | 2% |
| Month 3 after diagnosis | 1139 | $4,237 | 69% | 11% | 20% | 527 | $6,347 | 69% | 22% | 9% |
| Month 4 after diagnosis | 1121 | $3,216 | 62% | 11% | 27% | 515 | $5,918 | 74% | 16% | 10% |
| Month 5 after diagnosis | 1104 | $2,929 | 60% | 11% | 29% | 514 | $6,158 | 76% | 12% | 13% |
| Month 6 after diagnosis | 1086 | $2,422 | 52% | 14% | 35% | 511 | $4,266 | 73% | 10% | 17% |
| Monthly costs at the end of life | | |  |  |  |  |  |  |  |  |
| Month 6 before death | 344 | $6,367 | 67% | 11% | 23% | 151 | $4,511 | 57% | 9% | 34% |
| Month 5 before death | 358 | $6,155 | 71% | 9% | 20% | 152 | $5,059 | 60% | 12% | 29% |
| Month 4 before death | 373 | $8,001 | 75% | 10% | 15% | 152 | $5,936 | 73% | 9% | 18% |
| Month 3 before death | 389 | $8,111 | 77% | 9% | 14% | 164 | $7,458 | 76% | 11% | 14% |
| Month 2 before death | 410 | $9,075 | 82% | 9% | 9% | 170 | $7,793 | 84% | 7% | 9% |
| Final month of life | 445 | $20,670 | 92% | 5% | 3% | 181 | $20,088 | 93% | 4% | 4% |

*N/A: Not applicable due to zero/negative excess costs. Hospital-based costs included hospital admissions and ED presentations – ED presentations accounted for 6% of excess costs in the year and month pre-diagnosis, but in all other time periods and phases it accounted for <5% of excess costs (generally 2-3%).*

*Table I. Excess numbers of hospital days for colon and rectal cases diagnosed 2006-2013, by hospital type, for selected time periods.*

|  | **Colon cancer (n=1200)** | | | | | **Rectal cancer (n=546)** | | | | |
| --- | --- | --- | --- | --- | --- | --- | --- | --- | --- | --- |
| **Time period** | **No. of cases** | **No. of excess hospital days** | **Public hospital** | **Private hospital** | **% public hospital days** | **No. of cases** | **No. of excess hospital days** | **Public hospital** | **Private hospital** | **% public hospital days** |
| Phase of care |  |  |  |  |  |  |  |  |  |  |
| Initial | 1012 | 16.0 | 9.1 | 6.8 | 57% | 484 | 18.5 | 10.0 | 8.5 | 54% |
| Continuing (per year) | 905 | 1.7 | 1.0 | 0.7 | 60% | 443 | 2.0 | 1.4 | 0.7 | 67% |
| Terminal | 445 | 34.1 | 26.8 | 7.3 | 79% | 181 | 30.4 | 25.3 | 5.1 | 83% |
| Annual days relative to diagnosis |  |  |  |  |  |  |  |  |  |  |
| >1-2 years before diagnosis | 1200 | 0.0 | 0.1 | -0.1 | N/A | 546 | -0.3 | -0.2 | -0.1 | N/A |
| >0-1 year before diagnosis | 1200 | 1.1 | 0.7 | 0.3 | 70% | 546 | 0.7 | 0.7 | 0.0 | 93% |
| 0-1 year after diagnosis | 1200 | 19.6 | 12.5 | 7.1 | 64% | 546 | 20.4 | 12.4 | 8.0 | 61% |
| >1-2 years after diagnosis | 1011 | 4.3 | 2.9 | 1.4 | 67% | 484 | 3.8 | 2.7 | 1.1 | 71% |
| >2-3 years after diagnosis | 830 | 3.2 | 2.4 | 0.8 | 75% | 404 | 4.1 | 2.9 | 1.2 | 70% |
| >3-4 years after diagnosis | 632 | 3.4 | 2.5 | 0.9 | 73% | 318 | 3.4 | 2.6 | 0.8 | 77% |
| >4-5 years after diagnosis | 451 | 3.5 | 2.9 | 0.5 | 85% | 229 | 3.3 | 2.6 | 0.7 | 79% |
| Monthly days around diagnosis |  |  |  |  |  |  |  |  |  |  |
| Month 3 before diagnosis | 1200 | 0.0 | 0.0 | 0.0 | N/A | 546 | 0.0 | 0.0 | 0.0 | N/A |
| Month 2 before diagnosis | 1200 | 0.1 | 0.1 | 0.0 | N/A | 546 | 0.1 | 0.1 | 0.0 | N/A |
| Month 1 before diagnosis | 1200 | 0.8 | 0.6 | 0.2 | 79% | 546 | 0.4 | 0.3 | 0.1 | 68% |
| Month 1 after diagnosis | 1200 | 8.9 | 5.4 | 3.5 | 61% | 546 | 5.4 | 3.4 | 2.0 | 62% |
| Month 2 after diagnosis | 1163 | 3.8 | 2.7 | 1.1 | 70% | 535 | 3.7 | 2.4 | 1.3 | 65% |
| Month 3 after diagnosis | 1139 | 1.6 | 1.2 | 0.4 | 74% | 527 | 2.1 | 1.2 | 0.8 | 59% |
| Month 4 after diagnosis | 1121 | 1.1 | 0.8 | 0.3 | 73% | 515 | 1.9 | 1.0 | 0.9 | 52% |
| Month 5 after diagnosis | 1104 | 0.9 | 0.6 | 0.3 | 62% | 514 | 2.1 | 1.1 | 0.9 | 55% |
| Month 6 after diagnosis | 1086 | 0.6 | 0.3 | 0.3 | 49% | 511 | 1.5 | 1.0 | 0.5 | 64% |
| Monthly days at the end of life |  |  |  |  |  |  |  |  |  |  |
| Month 6 before death | 344 | 2.5 | 1.9 | 0.6 | 77% | 151 | 1.4 | 1.3 | 0.1 | 92% |
| Month 5 before death | 358 | 2.6 | 2.0 | 0.6 | 76% | 152 | 1.5 | 1.2 | 0.3 | 80% |
| Month 4 before death | 373 | 3.5 | 2.8 | 0.7 | 80% | 152 | 2.6 | 2.1 | 0.5 | 80% |
| Month 3 before death | 389 | 4.1 | 3.4 | 0.7 | 82% | 164 | 4.2 | 3.2 | 1.0 | 77% |
| Month 2 before death | 410 | 5.0 | 4.1 | 0.9 | 82% | 170 | 5.6 | 5.0 | 0.7 | 88% |
| Final month of life | 445 | 11.4 | 9.1 | 2.3 | 80% | 181 | 10.7 | 9.5 | 1.2 | 89% |

*N/A: Not applicable due to very small/negative excess days in hospital. Note: the “average” patient didn’t split their days in public/private as shown here, most were in only one type.*

*Table J. Mean excess costs for colon and rectal cases diagnosed 2006-2013, by spread of disease at diagnosis, for selected time periods.*

|  | **Colon cancer (n=1200)** | | | | **Rectal cancer (n=546)** | | | |
| --- | --- | --- | --- | --- | --- | --- | --- | --- |
| **Time period** | **Localised stage** | **Regional stage** | **Distant metastases** | **Unknown stage** | **Localised stage** | **Regional stage** | **Distant metastases** | **Unknown stage** |
| Phase of care | (n=398) | (n=492) | (n=243) | (n=67) | (n=187) | (n=221) | (n=90) | (n=48) |
| Initial | $36,077 | $56,774 | $79,437 | $30,887 | $49,072 | $73,159 | $82,116 | $34,353 |
| Continuing (per year) | $2,250 | $7,447 | $26,375 | $5,230 | $3,557 | $8,662 | $38,666 | $1,047 |
| Terminal | $69,304 | $75,831 | $81,186 | $39,025 | $68,491 | $66,077 | $68,817 | $67,084 |
| Annual costs around diagnosis |  |  |  |  |  |  |  |  |
| >1-2 years before diagnosis | $719 | -$447 | -$846 | $431 | $547 | -$88 | $381 | $2,110 |
| >0-1 year before diagnosis | $4,391 | $2,679 | $2,835 | $4,259 | $254 | $2,800 | $3,973 | $5,077 |
| 0-1 year after diagnosis | $38,522 | $60,284 | $82,785 | $32,273 | $51,158 | $75,377 | $81,987 | $31,749 |
| >1-2 years after diagnosis | $5,589 | $11,199 | $38,500 | $5,223 | $7,707 | $11,555 | $39,230 | $1,148 |
| >2-3 years after diagnosis | $4,005 | $10,893 | $32,218 | $3,794 | $5,744 | $13,094 | $30,874 | $11,589 |
| >3-4 years after diagnosis | $5,937 | $10,017 | $25,649 | $9,381 | $5,282 | $9,350 | $32,236 | $12,458 |
| >4-5 years after diagnosis | $4,652 | $7,578 | $12,465 | $13,529 | $1,807 | $9,368 | $36,069 | $7,035 |
| Monthly costs around diagnosis |  |  |  |  |  |  |  |  |
| Month 3 before diagnosis | $209 | $106 | -$102 | $396 | -$8 | $61 | $264 | -$508 |
| Month 2 before diagnosis | $297 | $564 | $310 | $466 | $147 | $198 | $328 | $901 |
| Month 1 before diagnosis | $1,412 | $2,122 | $3,540 | $1,236 | $760 | $1,079 | $1,156 | $2,216 |
| Month 1 after diagnosis | $21,188 | $29,834 | $30,270 | $15,308 | $15,922 | $19,380 | $18,190 | $9,068 |
| Month 2 after diagnosis | $9,126 | $9,594 | $11,234 | $7,165 | $12,460 | $12,171 | $11,508 | $6,540 |
| Month 3 after diagnosis | $2,463 | $4,637 | $7,610 | $1,691 | $5,158 | $6,989 | $9,910 | $3,062 |
| Month 4 after diagnosis | $1,359 | $3,365 | $6,642 | $2,839 | $3,693 | $8,696 | $6,979 | $2,087 |
| Month 5 after diagnosis | $1,249 | $3,012 | $6,501 | $2,485 | $3,431 | $8,345 | $9,120 | $3,350 |
| Month 6 after diagnosis | $659 | $2,343 | $7,340 | $1,002 | $2,937 | $4,677 | $6,536 | $4,013 |
| Monthly costs at the end of life |  |  |  |  |  |  |  |  |
| Month 6 before death | $5,076 | $5,074 | $8,787 | $4,226 | $3,384 | $5,589 | $4,928 | $745 |
| Month 5 before death | $3,606 | $6,069 | $8,001 | $2,067 | $4,403 | $4,126 | $5,269 | $9,736 |
| Month 4 before death | $4,952 | $7,111 | $10,937 | $2,296 | $4,626 | $4,644 | $4,457 | $22,508 |
| Month 3 before death | $7,272 | $8,116 | $9,661 | -$995 | $4,008 | $6,779 | $9,552 | $7,634 |
| Month 2 before death | $8,009 | $9,684 | $9,642 | $3,303 | $9,314 | $5,998 | $9,224 | $4,392 |
| Final month of life | $23,817 | $20,767 | $19,999 | $17,617 | $22,417 | $21,003 | $18,725 | $18,907 |

*Table K. Mean excess hospital days for colon and rectal cases diagnosed 2006-2013, by spread of disease at diagnosis, for selected time periods.*

|  | **Colon cancer (n=1200)** | | | | **Rectal cancer (n=546)** | | | |
| --- | --- | --- | --- | --- | --- | --- | --- | --- |
| **Time period** | **Localised stage** | **Regional stage** | **Distant metastases** | **Unknown stage** | **Localised stage** | **Regional stage** | **Distant metastases** | **Unknown stage** |
| Phase of care | (n=398) | (n=492) | (n=243) | (n=67) | (n=187) | (n=221) | (n=90) | (n=48) |
| Initial | 11.6 | 18.9 | 22.3 | 7.5 | 15.9 | 22.9 | 20.3 | 10.1 |
| Continuing (per year) | 0.3 | 2.5 | 3.0 | 2.2 | 0.7 | 2.5 | 8.1 | 0.2 |
| Terminal | 35.4 | 37.3 | 33.0 | 19.6 | 29.7 | 31.1 | 30.5 | 29.1 |
| Annual days relative to diagnosis |  |  |  |  |  |  |  |  |
| >1-2 years before diagnosis | 0.6 | 0.0 | -0.6 | -0.5 | -0.1 | -0.1 | -1.8 | 0.5 |
| >0-1 year before diagnosis | 1.5 | 1.1 | 0.1 | 1.1 | -0.2 | 0.5 | 2.4 | 1.5 |
| 0-1 year after diagnosis | 13.2 | 21.3 | 29.2 | 10.3 | 17.3 | 24.6 | 25.4 | 9.3 |
| >1-2 years after diagnosis | 1.6 | 4.9 | 12.4 | 1.0 | 2.7 | 2.9 | 13.7 | -0.2 |
| >2-3 years after diagnosis | 1.2 | 3.9 | 8.0 | 2.5 | 1.9 | 4.9 | 7.5 | 5.4 |
| >3-4 years after diagnosis | 1.6 | 4.2 | 7.3 | 3.3 | 0.9 | 3.5 | 12.0 | 4.7 |
| >4-5 years after diagnosis | 3.7 | 2.3 | 6.0 | 6.4 | 0.0 | 5.7 | 8.5 | 2.3 |
| Monthly days around diagnosis |  |  |  |  |  |  |  |  |
| Month 3 before diagnosis | 0.0 | 0.1 | -0.1 | 0.1 | 0.0 | 0.1 | 0.1 | -0.3 |
| Month 2 before diagnosis | 0.1 | 0.2 | 0.0 | 0.2 | 0.0 | 0.1 | 0.1 | 0.3 |
| Month 1 before diagnosis | 0.5 | 0.8 | 1.2 | 0.4 | 0.2 | 0.3 | 0.9 | 0.8 |
| Month 1 after diagnosis | 6.9 | 10.1 | 10.7 | 5.0 | 5.0 | 5.9 | 7.2 | 2.4 |
| Month 2 after diagnosis | 3.2 | 3.9 | 5.1 | 2.2 | 4.1 | 3.7 | 4.3 | 1.9 |
| Month 3 after diagnosis | 1.2 | 1.7 | 2.5 | 0.3 | 1.8 | 2.4 | 2.9 | 0.8 |
| Month 4 after diagnosis | 0.5 | 1.3 | 2.3 | 0.4 | 1.4 | 2.5 | 2.3 | 0.6 |
| Month 5 after diagnosis | 0.4 | 1.1 | 1.7 | 0.5 | 1.3 | 2.9 | 2.6 | 1.0 |
| Month 6 after diagnosis | 0.2 | 0.7 | 1.8 | 0.2 | 1.1 | 1.9 | 1.6 | 1.1 |
| Monthly days at the end of life |  |  |  |  |  |  |  |  |
| Month 6 before death | 2.1 | 2.2 | 2.8 | 3.1 | 0.5 | 2.1 | 1.5 | 0.3 |
| Month 5 before death | 1.9 | 3.1 | 2.6 | 1.4 | 1.1 | 1.8 | 1.4 | 2.5 |
| Month 4 before death | 3.7 | 3.3 | 3.9 | 1.1 | 2.0 | 2.7 | 2.2 | 5.6 |
| Month 3 before death | 4.2 | 4.2 | 4.5 | 0.0 | 2.7 | 3.4 | 5.2 | 5.8 |
| Month 2 before death | 5.4 | 5.4 | 4.8 | 2.3 | 5.5 | 5.3 | 6.4 | 3.7 |
| Final month of life | 10.7 | 11.8 | 11.5 | 10.3 | 12.3 | 10.6 | 11.2 | 6.2 |

*Fig A. Multivariable adjusted excess costs for colon and rectal cancer in the continuing phase of care.*

*Notes: The adjusted excess cost for a case in the reference category for all characteristics was $681 for colon cancer cases and $5201 for rectal. For each category of a characteristic, the estimate shown reflects the adjusted excess cost for a case with all other characteristics in the reference category. To allow non-negative regression, the models were constructed using an offset of +$50,000; this offset was then deducted to obtain the adjusted estimates shown in the figure.*

*Fig B. Multivariable adjusted excess hospital days for colon and rectal cancer in the continuing phase of care.*

*Notes: The adjusted excess hospital days for a case in the reference category for all characteristics is 1.4 days for colon cancer cases and 0.7 days for rectal. For each category of a characteristic, the estimate shown reflects the adjusted excess hospital days for a case with all other characteristics in the reference category. To allow non-negative regression, the models were constructed using an offset of +50 days; this offset was then deducted to obtain the adjusted estimates shown in the figure.*

*Fig C. Multivariable adjusted excess costs for colon and rectal cancer in the terminal phase of care.*

*Notes: The dashed vertical line is the adjusted excess cost for a case in the reference category for all characteristics (i.e. $103,223 for colon cancer cases and $87,736 for rectal). For each category of a characteristic, the estimate shown is the adjusted excess cost for a case with all other characteristics in the reference category. To allow non-negative regression, the models were constructed using an offset of +$50,000; this offset was then deducted to obtain the adjusted estimates shown in the figure.*

*Fig D. Multivariable adjusted excess hospital days for colon and rectal cancer in the terminal phase of care.*

*Notes: The dashed vertical line is the adjusted excess hospital days for a case in the reference category for all characteristics (i.e. 41 days for colon cancer, 38 days for rectal). For each category of a characteristic, the estimate shown is the adjusted excess days for a case with all other characteristics in the reference category. To allow non-negative regression, the models were constructed using an offset of +50 days; this offset was then deducted to obtain the adjusted estimates shown in the figure.*
