# Supplementary material for: Health system costs and days in hospital for colorectal cancer patients in New South Wales, Australia
Source: PLoS One. 2021 Nov 29;16(11):e0260088. doi: 10.1371/journal.pone.0260088 (PMC8629237; doi:10.1371/journal.pone.0260088)
Supplement: Attachment — Submitted filename: Response to Reviewers.docx [file pone.0260088.s004.docx]

**Editor**

**Comments**: Thank you for the submission to Plos One, and following on from our email correspondence, please accept my apologies for the delay in securing peer reviewers for this manuscript. Two reviews have been conducted, both are supportive of publication of this work. One review, by Reviewer 2, has identified a few areas that require slight changes in order to be clearer for the reader. Reviewer 1 requests: 'There are several analyses / tables presented, and I wondered about the rationale for looking at costs and hospital days in each of these ways – the 3-phase approach, the yearly 2-years pre-diagnosis to 5-years post-diagnosis data, as well as 3-month pre-diagnosis to 6-months post-diagnosis?', I think a clear statement about the rationale addressing this may also address some of reviewer 2's concerns and queries about the analytic approach. I hope these reviews are helpful in refining your paper.

**Response:** Thank you for the opportunity to revise and re-submit our paper. Thank you also to the editor and reviewers for your consideration and thoughtful suggestions, which have helped improve our work. We have added details and clarified areas as suggested, with a point-by-point response to all comments and details of changes provided below.

**Reviewer #1**

**Comment:** Thank you for the opportunity to review this excellent paper. The study uses a case-control design to investigate mean excess costs and days in hospital associated with a colon or rectal cancer diagnosis. The writing is very clear, methodology sound, and there are some interesting findings. The work is presented mainly as a preliminary paper to assist later modelling studies of cancer screening/prevention or treatment initiatives.

There are several analyses / tables presented, and I wondered about the rationale for looking at costs and hospital days in each of these ways – the 3-phase approach, the yearly 2-years pre-diagnosis to 5-years post-diagnosis data, as well as 3-month pre-diagnosis to 6-months post-diagnosis?

**Response:** Thank you for your feedback. As noted, the results will be used for future modelling studies, which may consider different post-diagnosis periods depending on the cancer control intervention to be evaluated. To allow for this study to inform a broad range of future evaluations, we wanted to make a broad range of different estimates available. We have added the following text to the methods section (page 10 of the version with tracked changes):

“The results for each of these time periods are presented to allow for use in future modelling studies, and to highlight the key time periods for resource use.”

**Comment:** For an international audience, it would be good to clarify briefly what kind of services (relevant to CRC) are provided by data from MBS, e.g. should note this includes GP attendance.

**Response:** We have added the underlined text in the methods section (page 6).

“The linked health records included reimbursements for government-subsidised prescription medicines in the Pharmaceutical Benefits Scheme (PBS), and outpatient and medical services (e.g. GP/specialist consultations, colonoscopies, CT scans, pathology tests) and some in-hospital procedures subsidised through the Medicare Benefits Schedule (MBS).”

**Comment:** The rationale for choosing socio-demographic/clinical factors associated with cost/days was not very well articulated. Results were then a little difficult to determine what was expected vs unexpected findings.

**Response:** We did not go into this study expecting certain differences in resource use, our aim was to report resource use and identify any associations within the data. The introduction includes the following, noting the factors are those potentially related to CRC incidence and treatment (page 6).

“We also aimed to disaggregate the estimates using several characteristics potentially related to health services use and/or CRC incidence, such as age, sex, health insurance status and accessibility of services.”

We have added the following text in the methods section (page 11).

“These characteristics were included due to their association with treatment and survival (27,28).”

**Comment:** It was helpful to see data not just on mean costs/hospital days, but also median/IQR and SD. Shows some pretty substantial variation, including into negative costs/days at some timeframes, vs controls (Table C) and suggests some cases – possibly a relatively small number – influencing costs. If there is room, it could be helpful to comment on this as it may be relevant for service providers to consider when organising CRC care.

**Response:** There are indeed some estimates with considerable variation. Random statistical variation for this large sample means there will be some cases with very high costs, and other cases will have controls with very high costs that result in very low or negative excess costs for the corresponding cases. However, the reasonably large sample size will make the estimates more robust to extreme outliers, as will the use of multiple matched controls for each case. To acknowledge this, we have added the following text in the limitations section (page 25).

“We focused on reporting means of excess costs, which can be influenced by extreme (high/low) values. However, the large sample size makes the estimates more robust to extreme outliers, limiting their influence, as does the use of multiple matched controls for each case. We have also reported other measures such as medians and standard deviations. Some groups of cases will have influence over the mean values, but this represents actual resource use and it is important to include these in estimates.”

**Comment:** Interesting findings regarding insurance status – it is suggested this could be due to different behaviours of people who have insurance, though services use might be related to how care is provided by public/private care providers.

**Response:** This is correct, and it is most likely a combination of both. We have undertaken a more in-depth investigation of the costing process and how the relevant healthcare records are generated. Our costing reflects the healthcare funder perspective, and it is possible that there are artefacts in the data that could lead to different levels of enumeration of costs by health insurance status. We undertook several sensitivity analyses, added the following text in the discussion regarding insurance, and removed the mention of insurance status in the abstract so as to not overstate the relationship.

Discussion (pages 22-23): “The differences in costs by health insurance could also be an artefact of how the costs were estimated, with potential for double-counting of some services in private hospitals that are also recorded in the MBS. After excluding all hospital-based MBS records, costs declined by ~5% across the three phases and most of the cost differences by insurance status were not statistically significant, while there were only minimal changes in the associations between costs and all other measured characteristics.”

Abstract (page 2): “Resources utilised (costs and days) in these phases increased with more advanced disease, comorbidities, and younger age~~, and private health insurance~~.”

**Reviewer #2**

**Comment:** Thank you for the opportunity to review this well considered manuscript. This will undoubtedly contribute to evaluations of screening and CRC management in Australia in the coming years.

The matched controls are described as ‘matched by age, sex, geography, smoking’ whilst the latter two variables offer some measure of correction for risk factors please clarify any precedents for this approach and acknowledge any potential shortcomings i.e. the limitation of obesity, alcohol consumption, or other SES related factors as known risk factors for CRC - This is pertinent since the subsequent stratification uses obesity as a classifier.

**Response:** Thank you for your thorough and helpful review. Our approach to matching controls to cases is based on three prior studies (now explicitly stated in the paper). These previous studies matched by age and sex only, and for this study we extended this to include geography and smoking status to further adjust for potential confounders, while achieving a high case-control matching ratio. While we agree that obesity, alcohol consumption and other SES related risk factors are important in the aetiology of colorectal cancer, to expand the number of characteristics for matching could lead to a lack of matched controls or the potential for over-fitting, either of which could lead to biases in the results. Instead, we adjusted for other potential confounders in the regression analysis, including comorbidities and body mass index (BMI). We have also compared cases and matched controls to verify similar distributions of area-level SES, private health insurance, BMI and self-reported health at recruitment (now added to as Supporting Information Table C). To clarify the approach in the manuscript, we added the following text to the Methods and Discussion and revised the abstract for clarity.

Methods (page 8 of the version with tracked changes): “The is an extension of the matching used in previous studies that estimated excess costs by age, sex and some geographical areas (19-21).”

Discussion (page 20): “Previous studies in England, the US, and New Zealand have reported the excess health system costs of care for CRC cases relative to cancer-free controls by age and sex (19-21). …”

Discussion (page 26): “Cases were matched to controls by age, sex, geography, and smoking status to estimate excess resource utilisation due to colorectal cancer. This extends the age-sex matching used in prior studies (19-21), but we did not explicitly control for other potential confounders through matching. There was little association between costs/days and BMI or socioeconomic level, so additionally matching on these factors would have little impact. The main associations with costs/days were for stage (not applicable to controls), age (a matching variable) and comorbidities. There were slightly more comorbidities recorded for cases than controls, but these mainly appeared or were recorded at hospital admissions in the 6 months leading up to diagnosis, and cases and controls had very similar self-reported health at baseline (Table C in S1 file). We did not include physical activity, alcohol consumption, or diet in the analysis, as these were beyond the scope of this study.”

Abstract (page 2): “… compared with those for cancer-free controls (matched by age, sex, geography, smoking) to estimate excess resources by phase of care, ~~stratified~~ analysed by sociodemographic, health, and disease characteristics.”

*Table C. Socioeconomic and health characteristics of colon and rectal cancer cases included in the study, compared with their matched controls.*

|  | **Colon (n=1200 cases)** | | **Rectal (n=546 cases)** | |
| --- | --- | --- | --- | --- |
|  | **% of cases** | **% of controls** | **% of cases** | **% of controls** |
| Area-level socioeconomic quintile |  |  |  |  |
| Most disadvantaged quintile | 25% | 24% | 23% | 22% |
| Quintile 2 | 21% | 22% | 22% | 23% |
| Quintile 3 | 19% | 18% | 20% | 19% |
| Quintile 4 | 15% | 16% | 17% | 16% |
| Least disadvantaged quintile | 18% | 18% | 16% | 18% |
| *Missing* | *2%* | *2%* | *2%* | *1%* |
| Health insurance status at baseline |  |  |  |  |
| Private insurance^a^ | 58% | 60% | 58% | 61% |
| Concession card | 27% | 25% | 24% | 21% |
| None | 13% | 13% | 16% | 15% |
| *Missing* | *3%* | *2%* | *3%* | *3%* |
| Body Mass Index |  |  |  |  |
| Normal/Underweight (<25kg/m^2^)^b^ | 35% | 35% | 33% | 33% |
| Overweight (25-<30) | 36% | 38% | 38% | 39% |
| Obese (≥30) | 22% | 19% | 23% | 21% |
| *Missing* | *7%* | *8%* | *5%* | *7%* |
| Baseline screening information^c^ |  |  |  |  |
| Ever had CRC screening | 45% | 52% | 29% | 50% |
| Ever had FOBT | 24% | 26% | 19% | 27% |
| Charlson comorbidity score |  |  |  |  |
| 0 | 83% | 88% | 87% | 90% |
| 1 | 10% | 7% | 8% | 5% |
| ≥2 | 8% | 5% | 5% | 5% |
| Self-reported health at baseline |  |  |  |  |
| Excellent | 13% | 12% | 11% | 14% |
| Very good | 33% | 35% | 37% | 34% |
| Good | 37% | 35% | 36% | 36% |
| Fair/Poor | 14% | 14% | 13% | 13% |
| *Missing* | *3%* | *4%* | *4%* | *3%* |

*^a^ In 2010, 55% of people in NSW and 53% of people in Australia had private health insurance (*[*https://www.apra.gov.au/quarterly-private-health-insurance-statistics*](https://www.apra.gov.au/quarterly-private-health-insurance-statistics)*; cited 29-Sep-2021).*

*^b^ “Underweight” (<18.5) accounted for ~1% of colon and rectal cases and controls.*

*^c^ The question asked about ever being screened or having FOBT and was not specific to the National Bowel Cancer Screening Program.*

*CRC: colorectal cancer; FOBT: faecal occult blood test.*

**Comment:** Line 165 onward: ‘This left 1% of admissions without an AR-DRG – almost all >2 years after diagnosis – and these were assigned a cost of $1500 for the first day (compared with single-day costs of ~$2200 for a colonoscopy and ~$1000 for rehabilitation) and $300 per additional day. A sensitivity test using double these assigned costs made <1% difference to the results.’ This comes across a little unclear? What do you mean? Is it that an alternative A or B costing was compared ... or that speculatively that a colonoscopy would be the rationale for any admission during this time? if so why ? Surely after a defined time frame it would not be an accepted standard that just because someone had a CRC that a scope would be indicated during an admission... surely a CT might be the first test of preference clinically?

Having read on it appeared this was a comparison ... therefore please frame the intro to the preceding sentence with the word 'either' and consider providing a rationale as to why a colonoscopy is the default test of preference at such a time point - in the absence of specific GI symptoms it would be more clinically likely that a CT would be ordered first as an inpatient if Hx of CRC were noted and the presenting condition thought in any way connected - ie they would want to exclude mets, rather than per se to re-examine the bowel itself +/- the chances that the individual had a rectal tumour and stoma placement etc...

**Response:** The costs listed were meant to provide a reference point to show that the hospitalisation costs being assigned in the absence of an AR-DRG are reasonable when compared to hospitalisation costs for patients undergoing common procedures. We have revised the text to clarify (page 9):

“This left 1% of admissions without an AR-DRG – almost all >2 years after diagnosis – and these were assigned a cost of $1500 for the first day ~~(compared with single-day costs of ~$2200 for a colonoscopy and ~$1000 for rehabilitation)~~ and $300 per additional day. To put these assumed costs into perspective, single-day hospitalisation costs were ~$2200 for a colonoscopy and ~$1000 for rehabilitation.”

**Comment:** Statistical analysis line 197: It might be useful if you had a small diagram to show this, such as the examples below.

Case	-----------------

C1	--------X

C2	----------------X

C3	----------------

Case	-----X

C1	-----X------------

C2	-----X------------

C3	-----X------------

**Response:** Thank you for the suggestion, we have added a version of this to the supporting information file (Figure A) and noted it in the manuscript (page 10).

“If the case was still alive and any of their matched controls died, the case and their remaining matched control(s) were included in any subsequent calculations, or they were censored if there were no remaining matched controls (Fig A in S1 File). …”

**Comment:** Line 207: 'Hospitalisations could span multiple time periods, so costs were apportioned across time periods using the proportion of days in each time period out of the total length of stay'. This is not super clear what that means... ? as in if a patient had a 3 week inpatient stay which crossed monthly cut points? or straddled a given year end? It would be helpful to clarify (based on the assumption that this work may form the basis for future updates of the work.)

**Response:** That is the correct meaning – for clarity we have modified the methods section as shown below (page 10).

“Costs were assigned to the relevant time period (phase/month/year relative to diagnosis/death) using the date of the MBS service or PBS supply or ED presentation. Hospitalisations could span multiple time periods, such as straddling the first and second year after diagnosis, or the initial phase and continuing phase. When this occurred, hospitalisation ~~so~~ costs were apportioned across time periods using the proportion of days in each time period out of the total length of stay for that hospitalisation.”

**Comment:** Line 211: How does the defined staging system compare to TNM Stage 1-4 ? and will that be useful for future work where the treatment indications are not by this staging system? Was TNM not available?

**Response:** The staging information in this study used the only staging data available. However, this staging provides a useful breakdown that can also be used in future modelling. The following text has been added to the limitations section (page 27).

“Also, the staging information available (localised, regional, distant metastases, unknown) does not include the detail provided by the TNM and other staging systems used in treatment guidelines (4), but it does give a meaningful and useful level of detail to differentiate between cancer patients that can be used in future modelling.”

**Comment:** Line 214: “…quintile of socioeconomic disadvantage of place of residence at baseline (22), health insurance status at baseline (private health insurance; healthcare concession card; none), body mass index (BMI: normal/underweight (<25kg/m2), overweight (25 to <30), obese (≥30)), comorbidities based on a modified Charlson Comorbidity Index (23) using hospital admissions up to 5 years prior to diagnosis (scores 0, 1, ≥2), and baseline self-reported CRC screening (any screening, and separately for FOBT).” *Were the controls also examined in this way for evidence of the effectiveness of their matching?*

**Response:** We thank the reviewer for the suggestion to add the comparison to the paper. As described in the response to a previous comment, we have added case-control baseline comparisons in the supporting information (Table C), with proportions of cases and controls for comorbidity scores, BMI, insurance, and self-reported CRC screening and self-reported health.

**Comment:** Line 259: “…but there were no records of subsidised use of the more recently introduced immunotherapies.” *Which given the time period of the dataset would be expected, right?*

**Response:** That is correct, and the manuscript has been revised to include this point (pages 12-13):

“… (bevacizumab from 2009, cetuximab from 2010), but there were no records of subsidised use of the more recently introduced immunotherapies, as expected based on the time period covered in the available data.”

**Comment:** Table 1: I would assume the layout will differ in print... but having the title layout not cross / doesn't hang over multiple pages makes it hard to read (at 6pm on a Friday...)

**Response:** This will depend on the Journal’s style and formatting. We will review this during the copy-editing process.

**Comment:** RE Private insurance coverage - How would this compare to the general Australian population? is that an expected level of private coverage.

**Response:** It is slightly higher in the study sample, with private insurance coverage for 60% of controls, 58% of cases, 55% of the population of NSW, and 53% of the population of Australia. We have added this to the Supporting Information (Table C, as described previously) and noted the difference in the limitations (page 26).

“There was also an over-representation of people with private health insurance, and people from regional and remote areas in the study sample …”

**Comment:** Table 1: 8% of colon cases had radiotherapy – presumably for lung/liver mets?

**Response:** That is a likely potential explanation, or possibly palliation for bone metastases, but we don’t have information on the indication for radiotherapy. Table F (was Table E) shows that 14% of colon cancer cases with distant metastases at diagnosis had radiotherapy. Three-quarters of the colon cancer cases who received radiotherapy had regional or metastatic spread at diagnosis. We do not have information on later disease progression to potentially explain the use of radiotherapy for those with localised disease at diagnosis, and a detailed investigation of the treatment types and timing was beyond the scope of this study.

**Comment:** Table 2 (high costs for colon cases with distant metastases): I am somewhat curious if this high level of spend is driven by chemo... since the % above suggested not... it seems hard to understand ... did this level of spend differ when you considered those with / without insurance?

**Response:** Table F (was Table E) shows that 62% of colon cancer cases with distant metastases at diagnosis had chemotherapy, compared with 54% of colon cancer cases with regional stage and 10% of colon cancer cases with localised stage, so there is higher chemotherapy use for those with advanced disease at diagnosis. The results from the regression analysis showed that colon cancer cases with private health insurance had higher costs than cases without private health insurance. However, among colon cancer cases with distant metastases at diagnosis, 68% of those with private insurance had chemotherapy, compared with 47% of concession card holders and 77% of uninsured cases, so it is potentially costs other than chemotherapy that are driving the association between costs and health insurance for cases with metastatic disease.

**Comment:** Results, terminal care phase: “The proportion of excess hospital days in public hospitals in this phase appeared to be higher than for other phases” As in private healthcare was less frequently used? I'd be curious to know how many died in hospital - and if the interaction with ?hospice care was explored?

**Response:** That is correct, private hospitals were less commonly used in the terminal care phase than in other phases. Unfortunately, hospices could not be identified in our data collection, only whether facilities were private or public. There was not detailed information about place of death, but of the cases who died, 66% had a hospital record that ended in death (added to the Results section). There were some records that noted death in a residential aged care facility (2% of deaths) or being dead on arrival at an emergency department (2%), but these fields were not completed for all decedents. In the Results section, we now explicitly state (page 18):

“Of cases who died, 66% had a record of dying in hospital.”

**Comment:** Discussion, paragraph 1: “We found substantial variation in costs and number of days in hospital by extent of disease at diagnosis…” Use stage instead of extent of disease, for consistency of wording.

**Response:** This change has been made throughout the manuscript.

**Comment:** Discussion, paragraph 1: “We found substantial variation in costs and number of days in hospital by extent of disease at diagnosis, highlighting cancer stage as a key factor in any analyses of CRC resource utilisation.” Yet, TNM staging not applied?

**Response:** As noted in the response to an earlier comment, TNM staging was not available for this study.

**Comment:** Discussion, paragraph 2: “…but only the English study provided a breakdown of costs by cancer stage (Stages 1-2 versus 3-4)” – which given how you report your data means that these are not comparable?

**Response:** The key point here was there are few detailed reports of costs. It has been noted that we are reporting costs according to the staging information available to us, and that this will not match all other staging classifications. We have revised the manuscript for clarity to now state (page 20):

“… but only the English study provided a breakdown of costs by cancer stage (Stages 1-2 versus 3-4) (20), and this is a topic with little reported information.”

**Comment:** Discussion, paragraph 2: “We were able to extend the Australian information to a population-based sample and include a wider range of care for CRC patients (e.g. extra GP/specialist consultations)” – this is the first mention of this.

**Response:** We have modified the introduction to include this information (page 5):

“Past studies have considered costs incurred by specific treatments (14-16), and our previous work ~~or~~ assessed total excess costs~~,~~ to capture~~ing~~ the overall burden on resources including services and/or care not flagged as being specific to cancer (e.g. extra GP/specialist consultations) (5).”

**Comment:** Discussion, paragraph 2: “Participants with “unknown” disease stage likely had less diagnostic work-up and less intensive treatment than other cases, as the classification of unknown stage indicates that insufficient information was available or provided to the cancer registry to assign stage, even if it was known to the treating clinician (28).” – Were there any checks for age of those with unknown stage - ie were they likely to be older or multimorbid? which would support this?

**Response:** There was very little difference in the proportions with unknown stage by age (6-8% across age groups) or comorbidity score (4-8% across comorbidity groups). We can give more information about the staging process. The cancer registry assigns stage using information received up to 4 months after the initial diagnosis date, including pathology results from samples taken during treatment. Table F (was Table E) shows cases with unknown stage were less likely to receive surgery (and other treatments), so these cases had fewer procedures that could generate staging information. We have revised the manuscript as shown below to clarify how diagnostic information received by the cancer registry up to 4 months after the initial diagnosis date is used for assigning stage.

Methods (page 11): “Cases were classified by cancer site (colon, rectal), NSWCR summary spread of disease at diagnosis (“stage”: localised; regional; distant metastases; unknown – based on notifications received by the NSWCR up to 4 months after diagnosis), …”

Discussion (page 21): “… the classification of unknown stage indicates that insufficient information was available or provided to the cancer registry up to 4 months after diagnosis to assign stage (e.g. pathology from a surgical procedure), …”

**Comment:** 4th paragraph in the Discussion on age (last sentence): 'A substantially smaller proportion of participants aged ≥75 years received chemotherapy or rectal surgery than was observed for those aged <75, and older participants may have had less diagnostic work-up'. How is this statement supported by the data... are they more represented in the stage unknown? just confused here, one can't be both staged and then have less diagnostic work up? They would have less surgical assessments, less ongoing CT monitoring, less ongoing blood tests ... follow up of care still in the time frame of your categorization of initial care ... but they would still have similar diagnostics to determine stage?

**Response:** We have modified the manuscript as suggested, and removed the mention of less diagnostic work-up (page 21).

“We found that a~~A~~ substantially smaller proportion of participants aged ≥75 years received chemotherapy or rectal surgery than was observed for those aged <75, and older participants may have had less intensive follow-up care ~~diagnostic work-up~~.”

**Comment:** Discussion: “Participants with colon cancer who did not live in a major city had lower costs than those in major cities during the initial and terminal care phases, perhaps due to treatment accessibility barriers, especially when travelling long distances and/or needing accommodation away from home.” – confusing - one might have thought that distance might have meant that care episodes were longer... but perhaps community based care services were more comprehensive for those in these areas?

**Response:** We have clarified this in the manuscript as shown below (page 22).

“Participants with colon cancer who did not live in a major city had lower costs than those in major cities during the initial and terminal care phases, perhaps due to treatment accessibility barriers limiting the amount of hospital-based or subsidised follow-up care, especially ~~when~~ for patients who needed to travel~~ling~~ long distances and/or find ~~needing~~ accommodation away from home.”

**Comment:** 6th paragraph: 'Compared with current smokers, ex-smokers who quit >15 years earlier had fewer hospital days during the initial and continuing phases. ' Please provide an effect size, or direct the reader to the section in the supplementary information.

**Response:** We have modified the manuscript to cite the results for the initial phase (page 23), and have removed the reference to the continuing phase as it was incorrectly left from an earlier version.

“~~Compared with current smokers,~~ Among rectal cancer cases, ex-smokers who quit >15 years earlier had fewer hospital days during the initial phase than current smokers (Fig 4; Table H in S1 file) ~~and continuing phases~~.”

**Comment:** In limitations you state: 'We did not include all possible resource-related information, with missing information on some non-admitted hospital costs (e.g. community-based care), treatment that may have been supplied by access programs (38), and numbers of hospital days did not include outpatient (non-admitted) procedures'. Have you clarified if colonoscopy is actually an outpatient procedure (as would be standard in the UK) - if so the attribution of a scope above and the $2200 shortfall should be acknowledged in the use of these costs for future researchers modelling as you suggest - clarification here would therefore be essential.

**Response:** Colonoscopies are performed as both an inpatient and an outpatient procedure in Australia. Previous work has shown that colonoscopies are well-captured in these datasets, along with other cancer treatments, and we have added this information to the Discussion section (page 27).

“We used detailed person-level information from a large prospective cohort study, and with the use of administrative health records, the resource utilisation data were not affected by recall bias, and provided comprehensive coverage of health services used (42,43).”

**Comment:** Discussion: “We could not evaluate differences in costs for cancers detected through screening compared with others, as we did not have data on whether participants were diagnosed via screening.” – You did have self-reported FOBT completion thou… right?

**Response:** As noted in the manuscript, we had self-reported FOBT/bowel check history at study baseline, but it was not necessarily related to the national population-wide screening program that was still in its relatively early stages of implementation. Given the timing from baseline to cancer diagnosis, any self-reported screening was unlikely to have been related to the CRC diagnosis. We have added the following text in the discussion (page 24):

“The self-reported FOBT/screening information was for activity prior to study baseline, and therefore is unlikely to refer to any screening episodes that led to the diagnosis of CRC.”

**Comment:** Discussion: “We hypothesise that CRC management would cost less and require less hospital time if CRC screening, via the NBCSP, had been fully rolled out during the period for which data were accrued, since effective screening is known to change the stage distribution in the population towards an earlier stage at diagnosis (36).” – But in the period prior to that when earlier diagnosis has not been fully realised, there is a latent incident case detection which should see a spike in cases... and costs (as they are of a more advanced stage ...) but over the period of your study have you seen any increased rate of detections? given that you reported 44% screening participation above its unlikely that the full benefits of the programme will be manifest, and that the stage distribution leaning to earlier detection will be stalled...

**Response:** We were not investigating total costs that would be influenced by overall numbers of cases, so we have modified the manuscript to clarify that we are describing resource use per case (page 24):

“We hypothesise that CRC management would cost less and require less hospital time per case if CRC screening, via the NBCSP, had been fully rolled out …”

**Comment:** Discussion: “There was also an over-representation of people from regional and remote areas in the study sample due to the 45 and Up Study’s sampling strategy, potentially underestimating some healthcare utilisation for colon cancers where those in major cities had higher resource utilisation.” – And or greater variation in insurance coverage.

**Response:** As noted above, we have modified the manuscript accordingly to state (page 25):

“There was also an over-representation of people with private health insurance, and people from regional and remote areas in the study sample …”

**Comment:** 2nd last paragraph: 'important to note that our study focused on direct healthcare costs incurred after a cancer diagnosis. '..... Why then in the discussion did you differentiate / suggest that diagnostic work up in older patients was likely… you couldn’t therefore determine that. Perhaps I wasn’t fully alert above – but a clear statement in the methods should indicate that no diagnostic costs are included – or at least that all costs are incurred after diagnosis.

**Response:** As noted above, we have adjusted the manuscript to clarify that diagnostic work-up can continue after the initial diagnosis, including information up to 4 months after the diagnosis date, so the costs can include diagnosis-related healthcare after the diagnosis date. Also, while we have focused on cost after diagnosis, we have reported costs in the months and years prior to diagnosis in Tables D, I and K, so pre-diagnosis costs have been included.

**Comment:** Conclusions: 'The results from this analysis will inform more detailed evaluations of the impact of CRC control interventions through costs and hospital requirements, and extend existing evaluations (9-13) to reinforce the benefit of interventions through changes in hospital utilization.' Why are there references in the conclusion? Surely that’s for the discussion… but also you’re pre-empting the results of future work that hasn’t been done here… Focus on your own conclusions.

**Response:** We have moved this section to the discussion as suggested.

**Comment:** 'This will help identify the best use of future healthcare resources and determine cost-effective strategies to reduce the CRC burden.' This is not a direct conclusion of this work, amend the previous sentence to consider some of the framing you wish… but otherwise move into the discussion.

**Response:** We have moved this section to the discussion.

*[Comments from Supplementary Information file]*

**Comment:** “The largest proportions by year of diagnosis for all CRC cases given targeted therapies were 16% and 6%, respectively, both for cases diagnosed in 2010, with virtually no records of these medications for cases diagnosed before 2008 (prior to their approval for subsidisation).” % of targeted therapy use right? please clarify in the opening of the sentence.

**Response:** It is the percent of all cases, not the percent of targeted therapy use. We have clarified this in the prior sentence as shown below. The dates of first use have also been added.

“Among all colon cancer cases, 10% had bevacizumab and 3% had cetuximab. For all rectal cancer cases, the proportions were 12% and 4%, respectively … with virtually no records of these medications for cases diagnosed before 2008 (prior to their approval for subsidisation, in 2009 and 2010, respectively).”

**Comment:** “Almost 30% of cases with metastatic disease at diagnosis had bevacizumab, compared with 10% of regional cases and 2% of localised cases.” Presumably this variation was attributable to insurance? Or was it that this is the % that went on to become metastatic and subsequently received them?

**Response:** There was very little difference in bevacizumab by insurance status. As per the text, the 30% refers to cases who had metastatic disease at diagnosis. We do not have information on later disease progression, and a detailed investigation of the treatment types and timing was beyond the scope of this study, so we have limited our reporting to these basic descriptive statistics by disease stage at diagnosis.

**Comment:** Table B; ARIA for study sample versus all NSW cases: was there a significance tests done here? would it be useful - not sure?

**Response:** We have not included significance tests here, as the sample size means that the differences resulting from the study sampling methods will be highly statistically significant. The manuscript includes a description of the over-sampling of people from regional and remote areas, and the discussion (pages 25-26) addresses the potential impact.

**Comment:** Table D: Clarify in table header row that it is for hospital days.

**Response:** The table (now Table E) has been modified accordingly.

**Comment:** Table E; 63% of metastatic colon cases had surgery: seems like a lot? ... is there any indication that this is hepatic surgery rather than primary disease? similarly i'm not sure what the XRT is for here... can you clarify what the standard care is at metastatic presentation ...

**Response:** As noted previously, a detailed investigation of treatment types is beyond the scope of this study. However a preliminary investigation of surgical procedures for metastatic colon cancer cases showed they were mostly “Right hemicolectomy with anastomosis” or “Low restorative anterior resection of rectum with coloanal anastomosis”, and were within a month or two of diagnosis. From the Australian treatment guidelines (reference 4), radiation therapy is not the standard of care for colon cancer cases during initial treatment, but it is noted as a potential treatment for recurrent disease. We do not have information on cancer recurrence and checking the indication for treatment for each case is beyond the scope of this study.

**Comment:** Table F: Consider re-formatting with shading (as per file).

**Response:** The tables for costs and hospital days (now Tables G and H) have been modified accordingly.

**Comment:** Table F: Why is 2010 the reference year?

**Response:** It was chosen as the reference year of diagnosis as it has the highest number of cases for both cancer types, and is in the middle of the study period.
